# Some Notes from a Personal Experience

**Published:** 1920-10-23

**Authors:** 


### THE VALUE OF EXPERIMENTS ON ANIMALS.

### Some Notes from a Personal Experience.

A particularly interesting series of notes on this subject by Sir Leonard Rogers, M.D., F.R.S., has been published by the Research Defence Society. The author, well known as the Professor of Pathology at the Medical College of Calcutta, has to his credit a great number of valuable researches, most of which have involved some degree of animal experiment. Also, generally speaking, he has been able to work entirely free from any restricting influence, such as that of the Act which is operative in England. Therefore, in making these extracts from his article it is possible to put before the reader a picture of what can, in these circumstances, be done and what has actually been done in bringing animal experiment to the service of pathological research, but particularly under essentially modern conditions. Occurrences before the days of the English Act are apt to be made use of in assessing its value at the present time, and quite rightly so. But it must be remembered that such evidence is not drawn from a modern scientific period. Sir Leonard Rogers' experience provides, on the other hand, a combination of the two circumstances—freedom of action and up-to-date investigation. As the review is made, it will be best to omit actual statement of the moral taught by each instance described, and to leave the reader to draw his own conclusions.

### Cattle and "Rinderpest."

While the author was in charge of the Civil Veterinary Department at Mukhtesar in the Kamoan Hills, an extensive series of experiments was carried out on cattle, involving the deliberate infection and sacrifice of some two hundred of them. The result of this work, together with that of Koch, Turner, and Kolle, led to the annual use of hundreds of thousands of successful preventive inoculations in India alone. The consequent continued and enormous reduction in the sufferings of cattle considered as a class, will probably go on as long as civilisation and human intelligence last. This is an interesting example, in that animals themselves, and not man alone, have gained directly by this work.

### The Cause of "Surra" in Horses.

For a number of years the problem of "surra," a fatal condition to horses in certain parts of India, defied all attempts at its solution. The causal organism, a trypanosome, had been discovered, but the essential point—namely, the mode of transference—still remained a mystery. Prevention under such conditions was inevitably merely guess-work, and unsuccessful at that. By means of a few experiments on dogs, rabbits, and rats, Sir Leonard Rogers was able to prove that the trypanosome, first discovered by Vet. Lt.-Col. Evans, was not transmitted by the mouth, unless the lips were actually excoriated at the time; whereas horse-flies fed on an infected animal could easily transmit the disease a few hours later to a healthy animal. The means of prevention and the benefit to the horse in those lands are obvious.

### The Study of Snake Venoms.

The work which led to the classification of snake-venoms is perhaps too well known to call for detailed description, but the author's discovery (necessarily at the expense of some "experimental" animals' lives) that a class known as viperine poisons caused death by paralysis of the vasomotor centre was at the time largely a new discovery. Indirectly this led to the preparation of an effective anti-venine against this type of venom also. Lamb and others in Bombay then produced a mixed serum with the power of neutralising both colubrine (the previously known type) and viperine poisons in the blood of a patient after he had been bitten by a poisonous snake, and this has ever since been made and extensively used in India.

Another particularly interesting example in this connection arose with the discovery that snake-venom might be injected into cats, and, in the absence of any antidotal serum, the lives of the animals saved by the simple operation of incising the site of injection of the poison and rubbing in permanganate crystals.

The last experiments are of very special interest from the point of view of the anti-vivisectionist campaign: because, so far back as 1874, Sir Lauder Brunton and Sir Joseph Fayrer had established the essential fact that permanganate solutions rapidly destroyed the activity of snake-venoms in test-tubes; but they were actually prevented from proceeding to the obvious next step—testing the practical efficiency of the methods on animals—by the passage of the Bill controlling experiments on animals.

As Sir Leonard remarks of his own experience, only ten cats and ten rabbits were required to save indirectly what must now be thousands upon thousands of human lives from one of the most terrible and tragic of all forms of death. So little did his cats actually suffer from these experiments that his only trouble, when visiting them after treatment, was that they all wanted their heads rubbed at the same time!

### Cholera and Leprosy.

The well-known hypertonic-saline treatment of cholera was largely worked out independently of animal work; but as it failed in certain extreme cases where a practically pure culture of the cholera vibrio was to be found throughout the whole bowel, a more powerful method had to be found, and here again the author, by means of the sacrifice of a very few rabbits, was able to establish the value of large doses of permanganate by the mouth. The estimated result was a further decrease of 10 per cent. in the mortality of the disease.

The treatment of leprosy by chaulmoogra oil had long been recognised, but was greatly handicapped in its efficiency by the difficulty of administering it in sufficient quantities. The author's contribution to a development, which is indeed still proceeding,

**The Value of Experiments on Animals**—(*continued*).

is amply illustrative of the indirect value of a few animals sacrificed. The question arose as to whether it would be safe to inject intravenously preparations of the unsaturated fatty acids of chaulmoogra oil. True oils, when so injected, produce fatal embolism. It was therefore clearly unjustifiable to carry this out unless its method could first be proved as safe upon animals. A few experiments on pigeons and rabbits, which caused no harm to them beyond a very temporary giddiness, showed that the intravenous route could be safely tried on man. Clinically the later results were remarkably satisfactory, and an undoubted step was made in solving the leprosy problem.

### Kala-azar.

To take one final illustration, it may be remembered that the disease associated with the Leishman-Donovan parasite had appeared in the form both of terrible epidemics and of sporadic cases in Assam and elsewhere. The problem of dealing with these latter (which practically means the control of the disease itself) could be solved only by the discovery of a cure for the disease. Animal experiments, and they alone, led up to the discovery that antimony salts are a cure for kala-azar, and, once this stage was reached, organised treatment and segregative measures assured the rest, and, as Sir Leonard Rogers says, animal experiment on a few rats in England has solved the problem of the appalling kala-azar epidemics of Assam, as well as of the more widely spread form of the disease in India and Northern Africa.

Similar instances can be given galore if the past records of medicine be searched, but recently, when this vital subject has come up for discussion, the tendency has been to choose examples of work at home and on home diseases, where manifestations are less terrible and extensive. Therefore there seems to be a double value in bringing to the public notice the more picturesque, but no less dramatic, successes achieved in the realms of tropical medicine.

